# Supplementary material for: Physicochemical Characterization and Drug Release Properties of Methyl-Substituted Silica Xerogels Made Using Sol–Gel Process
Source: Int J Mol Sci. 2021 Aug 25;22(17):9197. doi: 10.3390/ijms22179197 (PMC8430635; doi:10.3390/ijms22179197)
Supplement: Supplementary file 1 [file ijms-22-09197-s001.zip › ijms-1347516-supplementary.pdf]

A.1. Results

Table S1. Texture properties of the methyl substituted silica gels.

Sample (mol% MTES)	Hysteresis Type	Acid Catalysts	DFT (nm)	S_{BET}[a] (m^2/g)	S_{BET}[b] (m^2/g)	V_{micro} (cm^3/g)	V_{total} (cm^3/g)
**0**	H2a	A	4.72	448	494	0.133	0.373
	H3	B	32.65	291	283	0.110	0.562
**5**	H2a	A	3.06	563	608	0.168	0.385
	H2a	B	13.46	669	696	0.238	1.304
**10**	H2a	A	3.06	556	604	0.166	0.360
	H2a	B	7.3	777	813	0.266	0.945
**20**	H2a	A	3.06	588	627	0.221	0.335
	H2a	B	5.09	859	893	0.307	0.720
**30**	H2a	A	2.5	541	613	0.239	0.304
	H2a	B	4.89	930	976	0.324	0.728
**40**	H4	A	1.38	527	603	0.248	0.299
	H2a	B	5.09	1059	1115	0.356	0.931
**50**	H4	A	1.38	457	527	0.230	0.252
	H2a	B	5.29	851	893	0.303	0.799
**60**	H4	A	2.03	616	695	0.269	0.351
	H2a	B	4.89	895	938	0.332	0.704
**70**	H4	A	2.03	409	465	0.197	0.2378
	H4	B	2.03	694	722	0.275	0.482
**80**	H4	A	2.03	251	265	0.115	0.158
	H4	B	2.11	424	438	0.189	0.282

a – single point, b - multi point

Table S2. Average values of the pores and primary particles size obtained in small angle scattering measurements by fitting data with the unified exponential/power-law model.

**MTES content (%)**	**Diameter (nm)**			
	series A		series B	
	SANS	SAXS	SANS	SAXS
**0**	10.35 ± 0.16	8.11 ± 0.19	53.59 ± 6.02	30.68 ± 4.35
**5**	7.29 ± 0.09	6.34 ± 0.10	15.13 ± 0.35	11.39 ± 0.50
**10**	5.64 ± 0.08	5.36 ± 0.04	9.68 ± 0.17	8.37 ± 0.19
**20**	4.74 ± 0.11	4.65 ± 0.07	6.84 ± 0.14	6.34 ± 0.09
**30**	8.17 ± 1.06	5.59 ± 1.02	6.48 ± 0.18	5.85 ± 0.15
**40**	12.0 ± 2.21	8.60 ± 2.13	8.55 ± 0.31	7.59 ± 0.28
**50**	15.02 ± 2.28	28.08 ± 2.18	10.54 ± 0.27	10.45 ± 0.24
**60**	5.04 ± 0.63	5.38 ± 1.15	9.12 ± 0.45	9.28 ± 0.50
**70**	11.17 ± 5.33	7.59 ± 3.12	8.85 ± 0.86	8.48 ± 0.91
**80**	7.32 ± 1.32	6.89 ± 1.29	11.10 ± 4.25	9.69 ± 3.78

Table S3. XRD maxima position shift with the MTES mole percent

Sample	Position	FWHM	Height
**A0**	22.70	8.65	276.3
**A5**	22.65	8.65	260.7
**A10**	22.66	8.92	249.8
**A20**	22.61	9.42	226.5
**A30**	22.64	10.15	216.4
**A40**	22.57	10.87	211.9
**A50**	22.56	12.2	211.8
**A60**	22.47	12.06	209.2
**A70**	22.45	11.3	219.8
**A80**	22.34	11.43	216.3
**B0**	22.86	9.19	277.3
**B5**	22.70	9.06	240.9
**B10**	22.8	9.51	250.8
**B20**	22.81	10.01	244.2
**B30**	2.77	10.13	228.2
**B40**	22.72	9.98	207.2
**B50**	22.65	10.22	213.8
**B60**	22.51	10.78	215.5
**B70**	22.48	10.92	217.5
**B80**	22.32	11.07	211.4

Table S4. Quantitative analysis of ^{29}Si MAS NMR spectra.

**Sample**	**^{29}Si MAS NMR**											
	Q^4 (%)	Q^3 (%)	Q^2 (%)	Q (%)	T^3 (%)	T^2 (%)	T^1 (%)	T (%)	$Q^4 + T^3$ (%)	$Q^4 + T^3 + Q^3$ (%)	$Q^3+Q^2 + T^2$ (%)	$Q^2 + T^2$ (%)
**B0**	70.5	28.70	0.82	100	0	0	-	0	70.53	99.19	29.48	0.82
**B5**	63.3	32.30	1.51	97.20	T3+T2 = 2.82		-	2.82	66.16	98.49	33.84	1.51
**B10**	65.6	22.10	0	87.70	10.20	2.11	-	12.31	75.78	97.89	24.22	2.11
**B40**	43.6	14.80	0.62	58.9	26.50	14.51	-	41.05	70.11	84.86	29.88	15.13
**B60**	36.6	4.82	0	41.40	40.50	18.10	-	58.58	77.08	81.90	22.92	18.10
**B80**	17.4	1.58	0	19.90	61.18	18.90	0.91	80.07	78.61	80.19	20.47	18.89

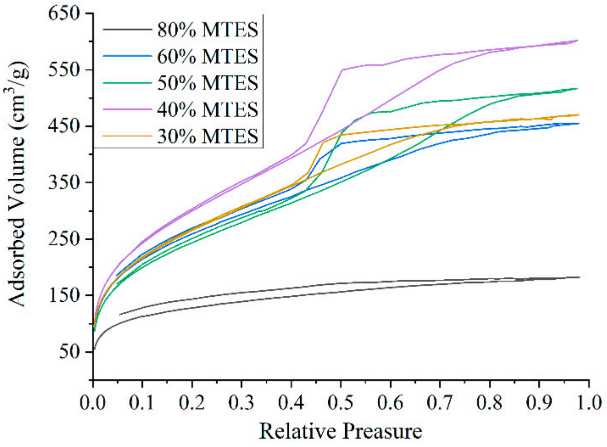

Figure S1. Evolution of nitrogen adsorption isotherms with 30-80% of MTES content.

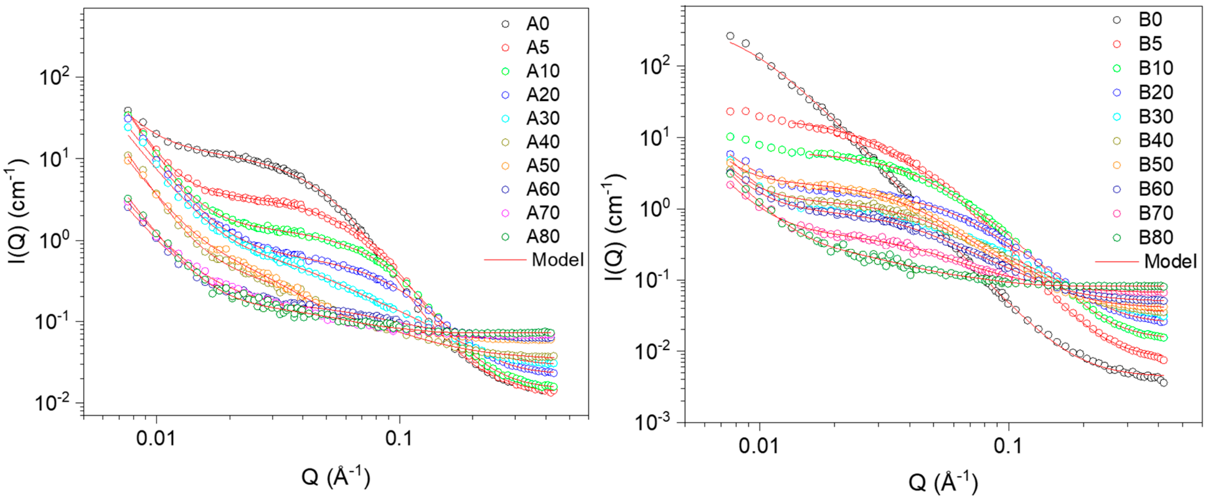

Figure S2. SANS profiles of series A (left) and B (right) as the amount of MTES in the reaction mixture increases from 5 % (A5/B5) up to 80 % (A80/B80). The SANS spectra of the TEOS silica precursor are also displayed for comparison (A0/B0). The applied model is also displayed as red lines.

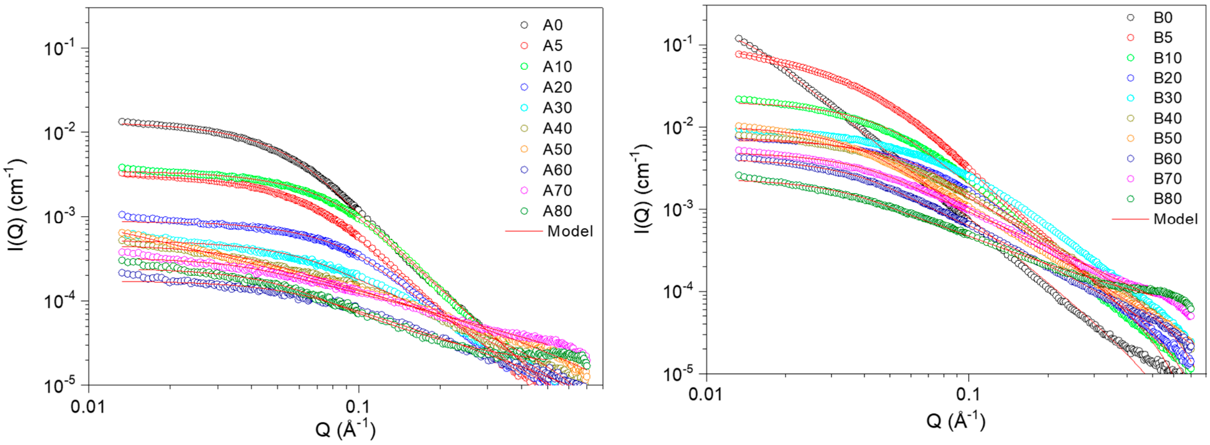

Figure S3. SAXS profiles obtained for series A (left) and B (right), as the amount of MTES in the reaction mixture increases from 5 % (A5/B5) up to 80 % (A80/B80). The SAXS spectra of the TEOS silica precursor are also displayed for comparison (A0/B0). The applied model is also displayed as red lines.

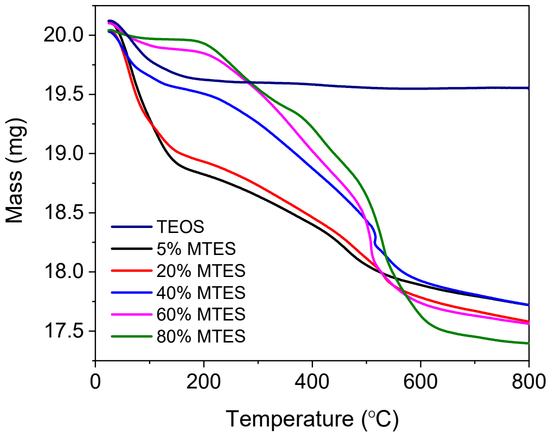

Figure S4. TG curves of the silica xerogels with different methyl content

**MTES content (%)**	**series A**		**series B**	
	**as-received**	**ground**	**as-received**	**ground**
0	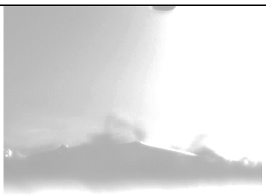	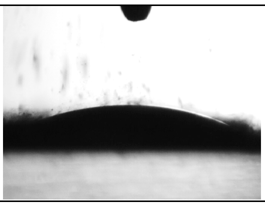	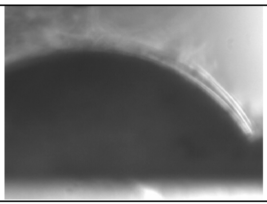	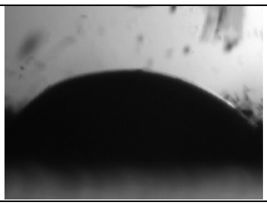

5	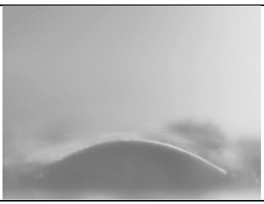	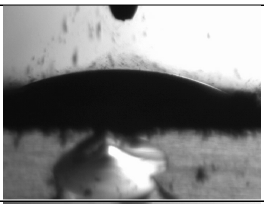	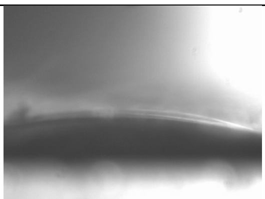	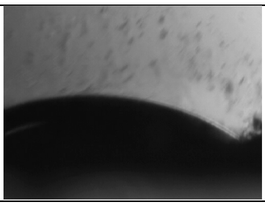
10	(no data)	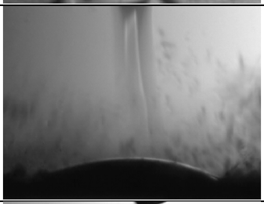	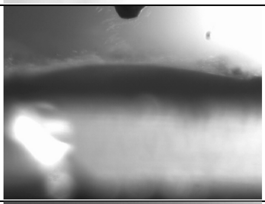	(no data)
20	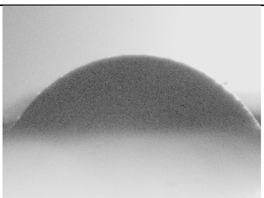	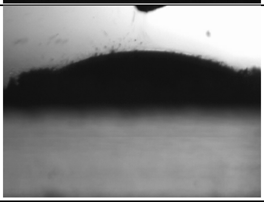	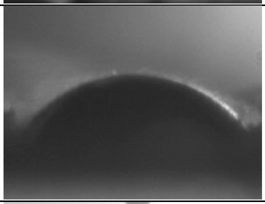	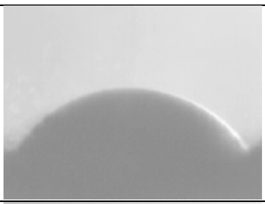
30	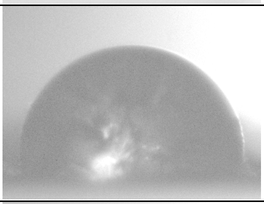	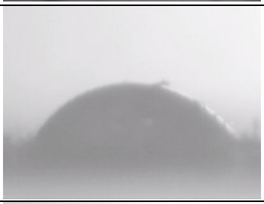	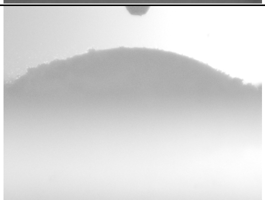	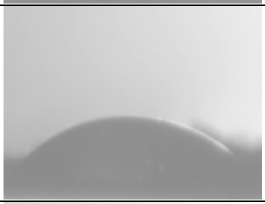
40	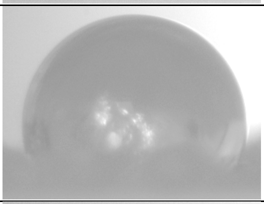	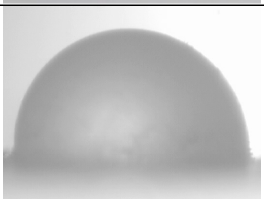	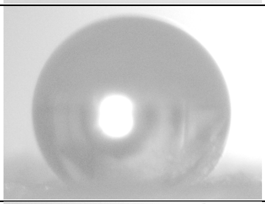	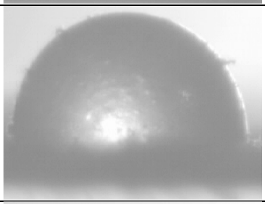
50	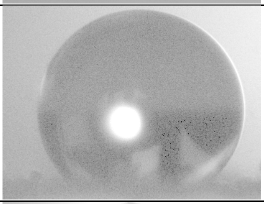	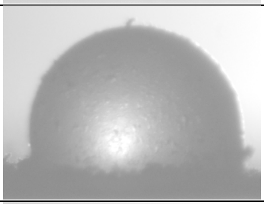	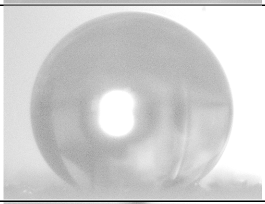	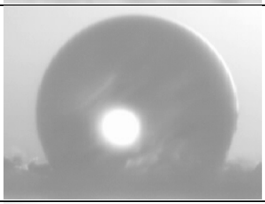
60	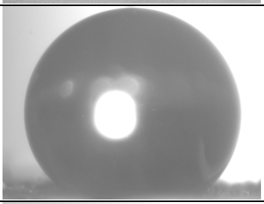	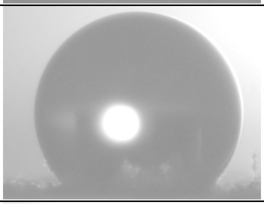	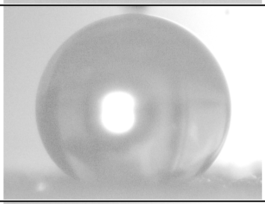	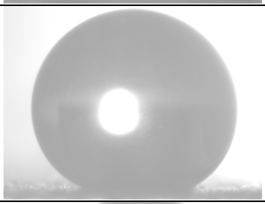
70	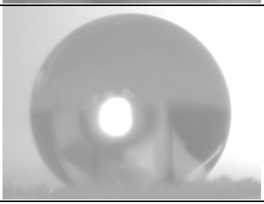	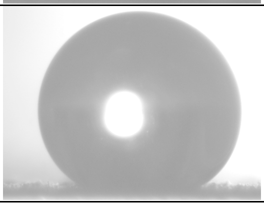	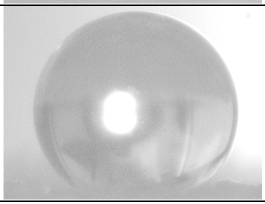	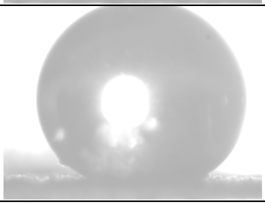

80	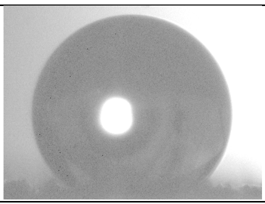	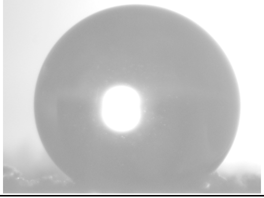	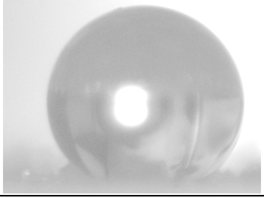	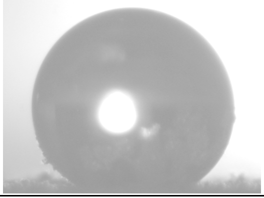

Figure S5. Representative contact angle images, one for each sample type, series A, respectively B, both as-received and ground.

Mathematical modelling of drug release

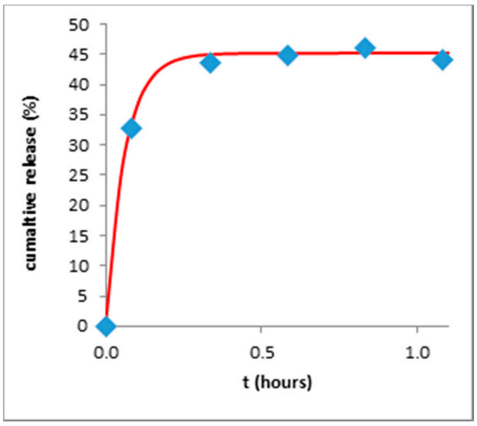

Figure S6. Fitted drug release curve of carrier A40 silica measured in HCl medium.
